# Nonpharmacological Methods to Reduce Pain During Active Labor in A Real-life Setting

**DOI:** 10.1055/s-0042-1759629

**Published:** 2023-03-06

**Authors:** Clarissa Bernardes de Oliveira Silva, Karine Mendonça Davi Rodrigues, Camila Zoldan, Roseli Mieko Yamamoto Nomura, Edward Araujo Júnior, Alberto Borges Peixoto

**Affiliations:** 1 Gynecology and Obstetrics Service, Hospital Universitário Mario Palmério, Universidade de Uberaba, Uberaba, MG, Brazil; 2 Department of Obstetrics, Escola Paulista de Medicina, Universidade Federal de São Paulo, São Paulo, SP, Brazil; 3 Department of Obstetrics and Gynecology, Universidade Federal do Triângulo Mineiro, Uberaba, MG, Brazil

**Keywords:** labor delivery, pain, intensity, visual analog scale, trabalho de parto, parto, intensidade, escala visual analógica

## Abstract

**Objective** To evaluate the association between pain intensity in the active phase of the first stage of labor with the use or not of nonpharmacological methods for pain relief in a real-life scenario.

**Methods** This was an observational cross-sectional study. The variables analyzed were obtained by a questionnaire with the mothers (up to 48 hours postpartum) to investigate the intensity of pain during labor using the visual analog scale (VAS). The nonpharmacological pain relief methods routinely used in obstetric practice were evaluated by consulting medical records. The patients were separated into two groups: Group I – patients who did not use nonpharmacological methods for pain relief and Group II –patients who used these methods.

**Results** A total of 439 women who underwent vaginal delivery were included; 386 (87.9%) used at least 1 nonpharmacological method and 53 (12.1%) did not. The women who did not use nonpharmacological methods had significantly lower gestational age (37.2 versus 39.6 weeks, *p* < 0.001) and shorter duration of labor (24 versus 114 min, *p* < 0.001) than those who used the methods. There was no statistically significant difference in the pain scale score using the VAS between the group that used nonpharmacological methods and the group that did not (median 10 [minimum 2–maximum 10] versus 10 [minimum 6–maximum 10] *p* = 0.334).

**Conclusion** In a real-life setting, there was no difference in labor pain intensity between the patients who used nonpharmacological methods and those who did not use them during the active phase of labor.

## Resumo

**Objetivo** Avaliar a associação da intensidade da dor na fase ativa da dilatação do parto em mulheres de acordo com a utilização ou não de métodos não farmacológicos para alívio da dor em cenário de vida real.

**Métodos** Trata-se de um estudo observacional de corte transversal. As variáveis analisadas foram obtidas através de questionário com as puérperas (até 48 horas pós-parto) investigando a intensidade da dor no parto pela escala visual analógica (EVA). As medidas não farmacológicas de alívio da dor, utilizadas rotineiramente na prática obstétrica, foram avaliadas pela consulta aos prontuários. As pacientes foram separadas em dois grupos: Grupo I – pacientes que não utilizaram medidas não farmacológicas para alívio da dor e Grupo II - pacientes que utilizaram estas medidas.

**Resultados** Foram incluídas 439 mulheres que tiveram parto vaginal, sendo que 386 (87,9%) utilizaram, pelo menos, uma medida não farmacológica e 53 (12,1%) não utilizaram. As mulheres que não utilizaram as medidas não farmacológicas apresentaram idade gestacional significativamente menor (37,2 *versus* 39,6 semanas, *p* < 0,001) e menor duração do trabalho de parto (24 *versus* 114 minutos, *p* < 0,001) quando comparadas às que utilizaram as medidas. Não houve diferença estatisticamente significativa na pontuação da escala da dor pela EVA de acordo com a categorização pelo uso ou não de métodos não farmacológicos (mediana 10 [mínimo 2–máximo 10] *versus* 10 (mínimo 6–máximo 10), *p* = 0,334].

**Conclusão** Em cenário de vida real, as pacientes submetidas aos métodos não farmacológicos não apresentaram diferença em relação à intensidade da dor quando comparadas às que não os utilizaram durante a fase ativa do trabalho de parto.

## Introduction

According to the International Association for the Study of Pain, pain is “an unpleasant sensory and emotional experience, associated with actual or potential tissue damage, or described in terms of such damage.” The intensity of pain is perceived differently by each subject and differs according to the individual's personality. Anxiety states potentiate pain, increase its perception, and decrease its tolerance, thereby generating muscle tension, which produces the “pain–tension–pain” circle. It is thus believed that several factors affect pain despite the existence of an organic cause. 1

Pain during labor is a visceral pain that occurs during the period of cervical dilation and distension of the lower uterine segment through the stimulation of nociceptors. It varies according to the genetic, psychological, and cultural characteristics of the woman and the birth process itself. Labor pain is part of human nature, and, unlike other acute and chronic painful experiences, it is not associated with disease but with the experience of giving birth to a new life. However, some women consider it to be the worst pain they ever felt and often greater than expected. 2

Some nonpharmacological interventions have been shown to help relieve labor pain and/or labor progression. Several studies indicate that Swiss ball exercises help with faster dilation, pain relief, and fetal descent. 3 4 5 The effectiveness of massage in reducing pain intensity has been demonstrated in several randomized trials. 6 7 8 A systematic review with 6 studies that included data on 326 women confirmed that massage significantly reduced the intensity of labor pain and also showed an improvement in the emotional experience of labor. 9 Another systematic review with data on 3,243 women showed that bathing reduced pain intensity in women with 8–9 cm cervical dilation, reduced the need for pharmacological analgesia, and shortened the duration of the first stage of labor. 10

Therefore, the aim of the present study was to evaluate the association between pain intensity in the active phase of the first stage of labor with the use of nonpharmacological methods for pain relief.

## Methods

This observational cross-sectional study was conducted between August 2019 and July 2021 at the Gynecology and Obstetrics Service of Mário Palmério University Hospital (MPHU), Uberaba – MG, Brazil. The study was approved by the Research Ethics Committee of the University of Uberaba (CAAE: 96383118.7.0000.5145). Informed consent was obtained from all participants included in the study.

Women who underwent vaginal delivery during the study period and who met the following criteria were included: pregnancy with a single live fetus, who progressed to vaginal delivery, and who did not receive pharmacological analgesia in labor. The women who did not wish to participate in the study and those who had difficulty in understanding the data collection instrument were excluded.

Recruitment took place in the postpartum period. The mothers who met the inclusion criteria were invited to participate in the study. During the first 48 hours postpartum, they were administered a structured questionnaire on the use of nonpharmacological methods for pain relief during labor. To assess the perception of pain during the active phase of labor, the visual analog scale (VAS) was used. 11 12 13 The VAS is a unidimensional instrument for assessing pain intensity. 11 It is a line with the ends being 0 and 10. One end of the line is marked with “no pain” and the other with “worst imaginable pain”. The patient is then asked to assess the pain and mark on the line that denotes the pain felt at that moment. Unidimensional instruments are advantageous in that they are easy, fast to apply, and inexpensive. 14 Intensity 0–2 was categorized as mild pain, 3–7 as moderate pain, and 8–10 as intense pain. The variables used to characterize the studied population were collected through the analysis of medical records. The information necessary for the research that could not be found in the medical records was researched through the analysis of prenatal card or directed anamnesis.

All patients included in the study were admitted to the obstetric center in the active phase of the first stage of labor. We consider active phase of labor the presence of cervical dilatation ≥ 6 cm associated with rhythmic, intense, and lasting uterine contractions. 15 We consider integral support from the medical team when the conduction of labor and delivery care was totally performed by medical residents and/or consultants. Obstetric nurses were part of the care team. Eventually, due to the occurrence of simultaneous vaginal deliveries/cesarean sections, the labor conduction of low-risk pregnancies was performed by the nursing team. The duration of labor was the time from the admission of the patient in the active phase of labor to fetal expulsion.

According to the institutional protocol, during admission, all patients were routinely informed about the possibility of nonpharmacological pain relief methods during assisted labor that could be requested at any time. Nonpharmacological pain relief methods are performed under the guidance and monitoring of the medical team, before the use of pharmacological methods. The application of nonpharmacological methods is performed continuously by resident physicians, nurses and, if present during labor, by doulas. Showering and exercising on the Swiss ball are provided, as well as guidance on breathing exercises, walking, and maternal mobility. In the private health network, women also have access to other nonpharmacological methods, such as acupuncture and transcutaneous electrical nerve stimulation, for pain relief. In the present study, the authors compared labor pain intensity among exposed or nonexposed parturients to the following nonpharmacological methods: showering, massage, transcutaneous electrical nerve stimulation, breathing exercises, relaxation techniques, walking, maternal mobility, and the Swiss ball.

Data were entered into a Microsoft Excel 2010 spreadsheet (Microsoft Corp., Redmond, WA, USA) and analyzed using IBM SPSS Statistics for Windows version 20.0 (IBM Corp., Armonk, NY, USA) and Prisma GraphPad 7.0 (GraphPad, San Diego, CA, USA)) software. The quantitative variables were initially assessed using the Kolmogorov-Smirnov test for normality. The variables that did not follow a normal distribution were expressed as median, minimum, and maximum values. The categorical variables were described as absolute frequencies and percentages and presented in tables. The chi-squared test was used to study the differences between groups regarding the categorical variables and their proportions, while the Mann-Whitney U test was used to assess differences between the groups regarding the continuous variables. The Spearman correlation test was used to evaluate the correlation between pain intensity and the analyzed variables. The significance level was set at *p* < 0.05 in all tests.

## Results

**Fig. 1:**
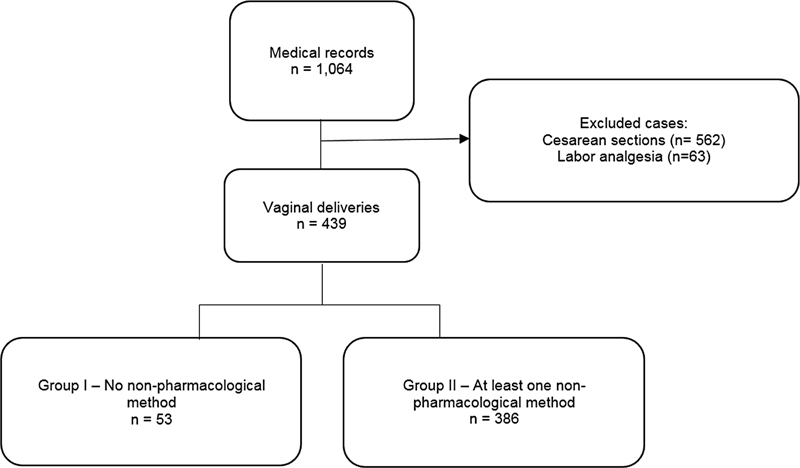
Flowchart of patient inclusion in the study.

There were 1,064 deliveries during the study period. The flowchart of patient inclusion is shown in Figure 1 . A total of 439 women who underwent vaginal delivery were included in the present study. The patients included in the study were divided into 2 groups: Group I ( *n* = 53), comprising patients who did not use any nonpharmacological method for pain relief during labor and Group II (n = 386), comprising patients who used at least one nonpharmacological method for pain relief.

A significant effect was observed between the groups and the gestational age at admission ( *p* < 0.001), duration of labor ( *p* < 0.001), and birthweight ( *p* = 0.019). The prevalence of at least one previous vaginal delivery was significantly higher in group I than in group II (71.7 versus 53.4%, *p* = 0.012) ( Table 1 ).

**Table 1:** Clinical characteristics of the study population

Variables	Group I ( *n* = 53)	Group II ( *n* = 386)	*p-value*
Age (years old)	24 (18–46)	23 (18–43)	0.772 ^†^
Ethnicity			0.815 ^∫^
White	26.4% (14/53)	32.4% (125/386)	
Black	20.8% (11/53)	18.4% (71/386)	
Mixed	52.8% (28/53)	49% (189/386)	
Asian	0% (0/53)	0% (0/386)	
Planned pregnancy	24.3% (13/53)	29.8% (115/386)	0.429 ^∫^
Acceptance of partner pregnancy	94.3% (50/53)	94.0% (363/386)	0.931 ^∫^
Type of health service			0.925 ^∫^
Public	96.2% (51/53)	95.6% (369/386)	
Private	3.8% (2/53)	4.4% (17/386)	
Initial maternal weight (Kg)	60 (35–84)	60 (30–133)	0.709 ^†^
Height (meters)	1.62 (1.50–1.72)	1.61 (1,43–1,88)	0.456 ^†^
BMI (Kg/m ^2^ )	23.1 (15.4–36.4)	23.2 (12,3–46,6)	0.747 ^†^
Smoking	3.8% (2/53)	8.0% (31/386)	0.270 ^∫^
Alcoholism	5.7% (3/53)	12.2% (47/386)	0.161 ^∫^
High-risk pregnancy	49.1% (26/53)	60.4% (233/386)	0.117 ^∫^
Illicit drug user	0% (0/53)	2.1% (8/386)	0.290 ^∫^
Number of pregnancies	2 (1–7)	2 (1–10)	0.007 ^†^
Parity	1 (0–5)	1 (0–9)	0.005 ^†^
Number of previous vaginal deliveries	1 (0–5)	1 (0–9)	0.008 ^†^
At least one previous vaginal delivery	71.7% (38/53)	53.4 % (206/386)	0.012 ^∫^
At least one previous cesarean section	7.6% (4/53)	10.9% (42/386)	0,632 ^∫^
Gestational age at admission (weeks)	37.2 (33-40)	39.6 (32-41,4)	< 0.001 ^†^
Number of prenatal care visits	7 (0–13)	8 (0–16)	0.090 ^†^
Duration of labor (minutes)	24.0 (0-210)	114,0 (0-5430)	< 0.001 ^†^
Birthweight (grams)	3,015 (545–4,030)	3,220 (1,415–4,355)	0.019 ^†^
Apgar score at 1 ^st^ min	8 (4–9)	8 (1–10)	0.360 ^†^
Apgar score at 5 ^th^ min	9 (7–10)	9 (5–10)	0.491 ^†^

Abbreviations: BMI, body mass index.

Group I: patients who did not use any nonpharmacological method for pain relief during labor; Group II: patients who used at least one nonpharmacological method for pain relief. Mann-Whitney ^†^ : median (minimum-maximum). Chi-squared ^∫^ : Percentage (absolute number/total number). *p* < 0.05.

There was no significant effect of the group on the pain intensity assessed by the VAS ( *p* = 0.334), as well as the degree of intensity reported by patients within the first 48 hours postpartum ( *p* = 0.830) ( Table 2 ).

**Table 2:** Pain intensity assessed by the visual analog scale (VAS) reported in the first 48 hours postpartum, among patients who did not use and who used at least one nonpharmacological method for pain relief during the active phase of the first stage of labor

	Group I ( *n* = 53)	Group II ( *n* = 386)	*p-value*
Pain intensity (VAS)	10 (6–10)	10 (2–10)	0.334 ^†^
Pain intensity degree			0.830 ^∫^
Mild	0% (0/53)	0.3% (1/386)	
Moderate	7.5% (4/53)	9.6% (37/386)	
Intense	92.5% (49/53)	90.2% (348/386)	

Abbreviation: VAS, visual analogue scale.

Group I: patients who did not use any nonpharmacological method for pain relief during labor; Group II: patients who used at least one nonpharmacological method for pain relief. Mann-Whitney ^†^ : median (minimum-maximum). Chi-squared ^∫^ : Percentage (absolute number/total number). *p* < 0.05.

**Fig. 2:**
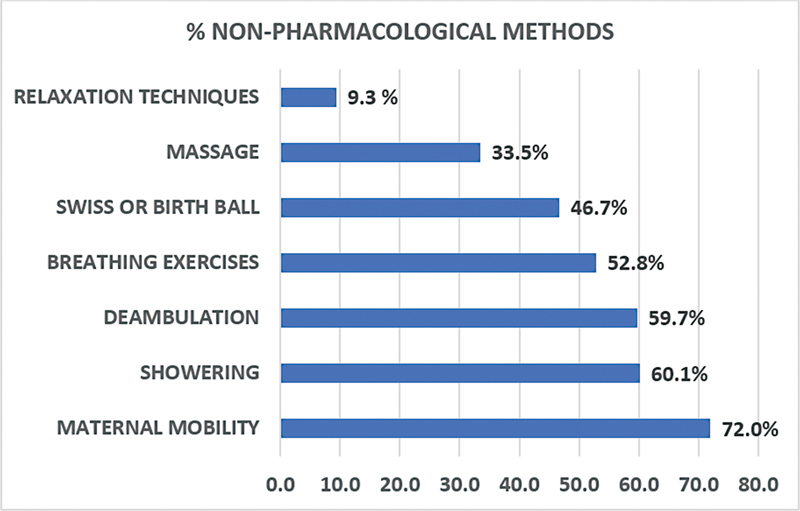
Prevalence of use of nonpharmacological methods for pain relief during the active phase of the first stage of labor in patients who underwent vaginal delivery.

The integral support of medical staff was present in 96.1% of all included cases in the study. The proportion of women who used nonpharmacological methods for pain relief during labor is shown in Figure 2 .

Table 3 shows the results of the association between pain intensity assessed by the VAS and the use of nonpharmacological methods for pain relief. There was no statistically significant difference in the VAS pain score between women who used nonpharmacological methods for labor pain relief and those who did not.

**Table 3:** Comparison of pain intensity perception score medians by the visual analog scale (VAS) between patients who used non-pharmacological pain relief methods during the active phase of labor and those who did not

	*n*	VAS	*p-value*
Maternal mobility			
Yes	316	10 (2–10)	0.110 ^†^
No	123	10 (5–10)	
Shower			
Yes	264	10 (3–10)	0.186 ^†^
No	175	10 (2–10)	
Walking			
Yes	262	10 (2–10)	0.126 ^†^
No	177	10 (5–10)	
Breathing Exercise			0.763 ^†^
Yes	232	10 (4–10)	
No	207	10 (2–10)	
Massage			
Yes	147	10 (4–10)	0.050 ^†^
No	292	10 (2–10)	
Relation techniques			
Yes	41	10 (6–10)	0.646 ^†^
No	398	10 (2–10)	
Swiss ball			
Yes	205	10 (2–10)	0.996 ^†^
No	234	10 (3–10)	
Any nonpharmacological method			0.249 ^†^
Yes	388	10 (2–10)	
No	51	10 (6–10)	

Abbreviation: VAS, visual analogue scale.

Mann-Whitney ^†^ : median (minimum-maximum), *p* < 0.05.

When evaluating the association of pain intensity in parturients who used at least one nonpharmacological method assessed by the VAS and history of previous vaginal delivery, there was no statistically significant difference in the VAS pain score between the groups (p= 0.732) ( Table 4 ).

**Table 4:** Pain intensity assessed by the visual analog scale (VAS), reported in the first 48 hours of postpartum, among patients that use at least one nonpharmacological method of pain relief and history of previous vaginal delivery

	At least one vaginal delivery ( *n* = 206)	Any previous vaginal delivery ( *n* = 180)	*p-value*
Pain intensity (VAS)	10 (2–10)	10 (3–10)	0.732 ^†^
Pain intensity degree			0.583 ^∫^
Mild	0.5% (1/206)	0.0% (0/180)	
Moderate	10.2% (21/206)	8.9% (16/180)	
Intense	89.3% (184/206)	91.1% (164/180)	

Abbreviation: VAS, visual analogue scale.

Considering the entire population included in the study, the Spearman correlation test was performed to assess the correlation between the degree of pain intensity during labor determined by the VAS and gestational age at admission, birthweight, duration of labor, and number of previous vaginal deliveries. No significant correlation was observed between the degree of pain intensity during the active phase of the first stage of labor and gestational age at admission ( *p* = 0.085), birthweight ( *p* = 0.899), number of previous vaginal deliveries ( *p* = 0.768), and duration of labor ( *p* = 0.159).

## Discussion

The aim of the present study was to assess the effect of the use of nonpharmacological methods for the relief of labor pain in a real-life setting by comparing the intensity of pain using the VAS between women who used these methods and those who did not. The use of nonpharmacological methods for labor pain relief did not lead to a difference in postpartum reporting of pain intensity by the VAS. Labor pain is a multidimensional subjective response and past experiences of pain may have an effect on the delivery. In a real-life setting, many aspects must be considered in the selection of practices when implementing pain relief protocols, including previous experiences.

The experience of labor pain as a natural physiological process is a widely accepted concept; however, this acceptance depends on the cultural environment in which women live and not all women fully accept the concept. In general, the use of nonpharmacological methods for pain management during labor is preferable because they have fewer adverse effects. They are simple methods that are easy to implement in care practice and are less expensive. However, if the characteristics of the population are not taken into consideration, they may not show the expected effectiveness. In a study that evaluated maternal satisfaction during childbirth, nonpharmacological techniques for pain relief, such as having a shower or a bath, massage, and exercises using a Swiss ball, were not associated with childbirth satisfaction. 16 Although this study did not assess pain intensity by objective methods, such as the VAS, the results are similar to those obtained in the present study, considering it was performed in a real-life setting.

Nonpharmacological methods of pain relief can be offered to women during labor and delivery because they can be effective. However, in obstetric practice, it is important to determine who women will benefit the most from specific techniques. For example, Yuksel et al. 17 demonstrated that breathing exercises during the second stage of labor are effective in reducing pain perception. This method was used in the present study; however, we did not limit the use of breathing exercise to the second stage of labor. In agreement with our results, Smith et al.. 18 in a meta-analysis, found low-quality evidence that massage provided greater reduction in pain intensity (measured using self-reported pain scales) than usual care during the first stage of labor (standardized mean difference [SMD]: 0.81; 95% confidence interval [CI]:- 1.06 − 0.56, 6 trials, 362 women). Conversely, 2 trials reported that the pain intensity during the second and third stages of labor showed reduction in pain scores in favor of massage (SMD: 0.98; 95%CI: - 2.23–0.26, 124 women; and SMD:1.03; 95%CI: - 2.17–0.11, 122 women). Pawale et al. 19 found that back massage was effective in reducing pain during the first stage of labor in primiparous women in comparison with those who received routine care. Therefore, the choice of best care depends on specific characteristics of the women.

The use of massage with local heat in the lumbosacral region considerably reduced the intensity of labor pain immediately and 30 min and 1 hour after the intervention. 20 These findings are in agreement with previous studies that recommend massage as an effective, noninvasive, and easy-to-use technique for relieving labor pain. In studies in which women were interviewed during labor, it is possible to observe the effects of the practice of nonpharmacological methods. However, it is possible that only the most intense pain is actually registered in the memory after the birth process. Because the present study was designed with the purpose of understanding the effect of the use of nonpharmacological methods in real-life practice, no interventions or investigations were performed during labor and the intensity of pain was assessed after the end of the process. The intensity of labor pain varies with each individual. The pain is moderate or severe, which is unbearable and increases the stress levels of the mother. Although labor pain has no underlying pathological process, it is associated with a painful experience and results in women worrying about how to avoid pain in future events.

In labor complicated by pain, the release of catecholamines further increases emotional stress and may delay the parturition process. In the present study, women who needed nonpharmacological methods for pain relief underwent a longer labor. The longer labor duration in parturients who needed nonpharmacological methods may be explained by the lower prevalence of at least one previous vaginal delivery. Encouraging the mother to embrace the natural birthing process by providing comforting techniques, such as patterned breathing, music, hydrotherapy, and relaxation, increases the production of endogenous endorphins that bind to receptors in the brain for pain relief. 20 21 22 In obstetric practice, the use of complementary practices can help in pain management.

Most women, especially nulliparous women, report fear of labor pain and this feeling becomes apparent in their recount of experiences. 23 In our study, there was no significant difference between the intensity of pain reported by the VAS and previous vaginal delivery. In a qualitative study, some women expressed that the pain is indescribable, some said that thought could not bear the pain, and others reported that were afraid to feel the pain and then progressed to a cesarean section and had two types of pains. 17 These reports demonstrate how labor pains are felt in general and that painful experiences are part of childbirth care practice. Skeide 24 argues that labor pains are shareable and should be shared in obstetric care practices and that labor pains are not essentially destructive.

Unfortunately, in several maternity hospitals in Brazil, both public and private, the participation of doulas in obstetric care is not yet completely widespread. For this reason, we aimed to evaluate the association between pain intensity in the active phase of the first stage of labor with use or not of nonpharmacological methods for pain relief in a real-life scenario **,** in which the conduction of delivery and application of nonpharmacological methods is still performed by the medical team.

The presence of doulas is regulated in our service; however, perhaps due to lack of knowledge or lack of purchasing power of patients, the presence of these professionals during the labor is still very low. In our study, we did not survey the prevalence of doulas during the labor care, neither the comparation of intensity of pain when nonpharmacological methods were applied by the skills of different professionals. In our study, the support of the medical and nursing team for the application of nonpharmacological methods was continuous, in an attempt to ensure better effectiveness of the method used for pain relief during conduction of delivery.

The limitations of the present study included a smaller sample of women who did not use nonpharmacological methods in obstetric practice, and the reasons for not using these methods could be associated with the characteristics of these pregnancies. It is recommended that similar studies in real-life settings be performed in the future to compare specific groups of nulliparous and multiparous women during labor and 1 year postpartum. The studies could also include different approaches to identify the moments when pain is more relevant and the role of the selected practices. Therefore, the comparison of different nonpharmacological pain relief methods allows suggesting a method that may be suitable for most women in relieving labor pain.

## Conclusion

In summary, in the present real-life practice study, there was no association between the use of nonpharmacological methods for labor pain relief and reduction of pain intensity experienced by the parturients. Other techniques, in addition to those described, may be implemented in the future to widen the range of options for reducing labor pain in clinical practice. Furthermore, the comparison of the pain relief and reduction in the pain intensity, when nonpharmacological methods are performed by different professionals, is also necessary for better assessment of efficacy of these methods in obstetrical practice.

## References

[1] Raja S N Carr D B Cohen M Finnerup N B Flor H Gibson S The revised International Association for the Study of Pain definition of pain: concepts, challenges, and compromises Pain 2020 161 09 1976 1982 10.1097/j.pain.0000000000001939 32694387 PMC7680716

[2] Lowe N K The nature of labor pain Am J Obstet Gynecol 2002 186(5, Suppl Nature):S16 S24 10.1067/mob.2002.121427 12011870

[3] Gau M L Chang C Y Tian S H Lin K C Effects of birth ball exercise on pain and self-efficacy during childbirth: a randomised controlled trial in Taiwan Midwifery 2011 27 06 e293 e300 10.1016/j.midw.2011.02.004 21459499

[4] Leung R W Li J F Leung M K Fung B KY Fung L CW Tai S M Efficacy of birth ball exercises on labour pain management Hong Kong Med J 2013 19 05 393 399 10.12809/hkmj133921 23878201

[5] Makvandi S Latifnejad Roudsari R Sadeghi R Karimi L Effect of birth ball on labor pain relief: A systematic review and meta-analysis J Obstet Gynaecol Res 2015 41 11 1679 1686 10.1111/jog.12802 26419499

[6] Chang M Y Chen C H Huang K F A comparison of massage effects on labor pain using the McGill Pain Questionnaire J Nurs Res 2006 14 03 190 197 10.1097/01.jnr.0000387577.51350.5f 16967401

[7] Taghinejad H Delpisheh A Suhrabi Z Comparison between massage and music therapies to relieve the severity of labor pain Womens Health (Lond Engl) 2010 6 03 377 381 10.2217/whe.10.15 20426604

[8] Silva Gallo R B Santana L S Jorge Ferreira C H Marcolin A C Polineto O B Duarte G Massage reduced severity of pain during labour: a randomised trial J Physiother 2013 59 02 109 116 10.1016/S1836-9553(13)70163-2 23663796

[9] Smith C A Levett K M Collins C T Jones L Massage, reflexology and other manual methods for pain management in labour Cochrane Database Syst Rev 2012 02 CD009290 10.1002/14651858.CD009290.pub2 22336862

[10] Davim R M Torres G V Dantas J C Melo E S Paiva C P Vieira D [Showering as a non-pharmacological strategy to relief the parturients pain] Rev Eletrônica Enferm 2008 10 03 600 609 10.5216/ree.v10.46588

[11] Bijur P E Silver W Gallagher E J Reliability of the visual analog scale for measurement of acute pain Acad Emerg Med 2001 8 12 1153 1157 10.1111/j.1553-2712.2001.tb01132.x 11733293

[12] Wadhwa Y Alghadir A H Iqbal Z A Effect of antenatal exercises, including yoga, on the course of labor, delivery and pregnancy: a retrospective study Int J Environ Res Public Health 2020 17 15 5274 10.3390/ijerph17155274 32707830 PMC7432001

[13] Czech I Fuchs P Fuchs A Lorek M Tobolska-Lorek D Drosdzol-Cop A Pharmacological and non-pharmacological methods of labour pain relief-establishment of effectiveness and comparison Int J Environ Res Public Health 2018 15 12 2792 10.3390/ijerph15122792 30544878 PMC6313325

[14] Martinez J E Grassi D C Marques L G Analysis of the applicability of different pain questionnaires in three hospital settings: outpatient clinic, ward and emergency unit Rev Bras Reumatol 2011 51 04 299 303, 308 21779706

[15] Obstetric care consensus no. 1: safe prevention of the primary cesarean delivery Obstet Gynecol 2014 123 03 693 711 10.1097/01.AOG.0000444441.04111.1d 24553167

[16] Lopes F Nakamura M U Nomura R MY Women's satisfaction with childbirth in a public hospital in Brazil Birth 2021 48 02 251 256 10.1111/birt.12534 33543497

[17] Yuksel H Cayir Y Kosan Z Tastan K Effectiveness of breathing exercises during the second stage of labor on labor pain and duration: a randomized controlled trial J Integr Med 2017 15 06 456 461 10.1016/S2095-4964(17)60368-6 29103415

[18] Smith C A Levett K M Collins C T Dahlen H G Ee C C Suganuma M Massage, reflexology and other manual methods for pain management in labour Cochrane Database Syst Rev 2018 3 03 CD009290 10.1002/14651858.CD009290.pub3 29589380 PMC6494169

[19] Pawale M P Salunkhe J A Effectiveness of back massage on pain relief during first stage of labor in primi mothers admitted at a Tertiary care center J Family Med Prim Care 2020 9 12 5933 5938 10.4103/jfmpc.jfmpc_1189_20 33681022 PMC7928123

[20] Kaçar N Özcan Keser N Comparison of the effect of mechanical massage and warm mechanical massage application on perceived labor pain and childbirth experience: A randomized clinical trial Eur J Midwifery 2021 5 5 10.18332/ejm/132883 33655203 PMC7910811

[21] Boateng E A Kumi L O Diji A K Nurses and midwives' experiences of using non-pharmacological interventions for labour pain management: a qualitative study in Ghana BMC Pregnancy Childbirth 2019 19 01 168 10.1186/s12884-019-2311-x 31088408 PMC6518741

[22] Kumarilohar S Deepak Y Vaishnav A An experimental study to assess effectiveness of music therapy in labor pain reduction among primigravida Mothers in first stage of labor in selected hospitals at Udaipur, Rajasthan IOSR J Nurs Health Sci 2018 7 04 28 32

[23] Khatony A Soroush A Andayeshgar B Saedpanah N Abdi A Attitude of primiparous women towards their preference for delivery method: a qualitative content analysis Arch Public Health 2019 77 38 10.1186/s13690-019-0364-y 31452882 PMC6700977

[24] Skeide A Experiences as actors: labor pains in childbirth care in Germany Med Anthropol 2021 40 05 446 457 10.1080/01459740.2020.1860963 33400594

